# Experimental Infection of Domestic Pigs with African Swine Fever Virus Isolated in 2019 in Mongolia

**DOI:** 10.3390/v14122698

**Published:** 2022-12-01

**Authors:** Chester D. McDowell, Dashzeveg Bold, Jessie D. Trujillo, David A. Meekins, Cassidy Keating, Konner Cool, Taeyong Kwon, Daniel W. Madden, Bianca L. Artiaga, Velmurugan Balaraman, Ulaankhuu Ankhanbaatar, Batsukh Zayat, Jamie Retallick, Kimberly Dodd, Chungwon J. Chung, Igor Morozov, Natasha N. Gaudreault, Jayme A. Souza-Neto, Jürgen A. Richt

**Affiliations:** 1 Department of Diagnostic Medicine/Pathobiology, College of Veterinary Medicine, Kansas State University, Manhattan, KS 66506, USA; 2 State Central Veterinary Laboratory, Ulaanbaatar 17024, Mongolia; 3 Institute of Veterinary Medicine, Mongolian University of Life Science, Ulaanbaatar 17024, Mongolia; 4 Veterinary Diagnostic Laboratory, College of Veterinary Medicine, Michigan State University, Lansing, MI 48824, USA; 5 Proficiency and Validation Service Section, Foreign Animal Disease Diagnostic Laboratory, Animal and Plant Health Inspection Services, United States Department of Agriculture, Plum Island Animal Disease Center, Greenport, NY 11944, USA

**Keywords:** ASFV, ASF, Mongolia, virulence, acute disease, genotype II, domestic pigs

## Abstract

African swine fever (ASF) is an infectious viral disease caused by African swine fever virus (ASFV), that causes high mortality in domestic swine and wild boar (*Sus scrofa*). Currently, outbreaks are mitigated through strict quarantine measures and the culling of affected herds, resulting in massive economic losses to the global pork industry. In 2019, an ASFV outbreak was reported in Mongolia, describing a rapidly progressing clinical disease and gross lesions consistent with the acute form of ASF; the virus was identified as a genotype II virus. Due to the limited information on clinical disease and viral dynamics within hosts available from field observations of the Mongolian isolates, we conducted the present study to further evaluate the progression of clinical disease, virulence, and pathology of an ASFV Mongolia/2019 field isolate (ASFV-MNG19), by experimental infection of domestic pigs. Intramuscular inoculation of domestic pigs with ASFV-MNG19 resulted in clinical signs and viremia at 3 days post challenge (DPC). Clinical disease rapidly progressed, resulting in the humane euthanasia of all pigs by 7 DPC. ASFV-MNG19 infected pigs had viremic titers of 10^8^ TCID_50_/mL by 5 DPC and shed virus in oral secretions late in disease, as determined from oropharyngeal swabs. Whole-genome sequencing confirmed that the ASFV-MNG19 strain used in this study was a genotype II strain highly similar to other regional strains. In conclusion, we demonstrate that ASFV-MNG19 is a virulent genotype II ASFV strain that causes acute ASF in domestic swine.

## 1. Introduction

African Swine Fever (ASF) is an infectious viral disease that causes high mortality in domestic swine and wild boar (*Sus scrofa*). Outbreaks of the disease have devastating economic impacts on affected regions and threaten swine health and the swine industry worldwide. No commercial vaccine or treatment is currently available, except for a live-attenuated vaccine called NAVET-ASFVAC that was recently (June 2022) licensed for restricted use only in Vietnam [1,2], however currently suspended by Vietnam’s Minister of Agriculture and Rural Development (MARD) due to improper use that resulted in the death of vaccinated pigs. Therefore, the current control measures for ASF primarily rely on quarantine and biosecurity, animal movement restrictions, and the depopulation of infected/exposed animals [3].

The causative agent of ASF is African Swine Fever virus (ASFV), which belongs to the *Asfarviridae* family, genus *Asfivirus* [4,5]. The virus particle encapsulates a 170–192 kilobase (kB) double-stranded DNA genome encoding for more than 150 genes [6,7,8,9]. Currently, 24 genotypes have been identified with varying virulence between and within genotypes [10,11]. Historically, ASFV was restricted to Sub-Saharan African countries until outbreaks emerged between the 1950s and 1980s in Europe, Russia and South America; by the 1990s, ASFV was confined to Africa and Sardinia, where it remained endemic [4]. In 2007, an outbreak in domestic pigs in the country of Georgia resulted in the spread of ASFV into eastern Europe and throughout Russia, where it became established in wild boar populations [12,13,14]. In 2018, China reported the first outbreak in Asia. By 2019, ASFV was reported in neighboring countries, including Cambodia, Laos, Mongolia, Myanmar, the Philippines, and Vietnam, as well as the Korean peninsula [15].

In January of 2019, the first case of ASFV was reported in a backyard herd consisting of 38 pigs in the Bulgan province of Mongolia [16]. Following the index outbreak, subsequent outbreaks were confirmed in seven Mongolian provinces. Due to the rapid implementation of control measures, which included surveillance, movement restrictions in the affected districts, culling of the affected herds, and a temporary ban on the importation of pork products, the outbreak was declared over by April 2019 [16]. The outbreak resulted in the loss of 2862 pigs, equivalent to 10% of the total Mongolian pig population [16,17]. Initial molecular characterization of Mongolian field isolates determined that the circulating strain was a genotype II strain that was highly similar to other genotype II viruses found in China and Russia based on the major capsid protein (p72) and envelope protein (p54) sequences, as well as the central hypervariable region (CVR) of B602L (viral chaperone) [17].

Information on the clinical signs and progression of the ASFV Mongolia/2019 (ASFV-MNG19) strain is limited to observations from the field, describing clinical signs and gross pathological lesions that are consistent with an acute form of ASF [17]. In this study, we investigated the virulence and clinical progression of ASFV-MNG19 in a controlled experimental setting with domestic swine.

## 2. Materials and Methods

### 2.1. Cell Culture and Challenge Material Preparation

ASFV-MNG19 was isolated from the kidney of an infected pig from the Tov province of Mongolia during the 2019 outbreak. A tissue homogenate was prepared from the kidney and shipped to Kansas State University (KSU) via USDA-APHIS. All experiments involving ASFV-MNG19 were conducted in the BSL-3 laboratory at the Biosecurity Research Institute (BRI) at KSU in Manhattan, KS. ASFV-MNG2019 was propagated in T-75 cm^2^ flasks on near confluent primary swine pulmonary alveolar macrophages (PAM). Inoculated cell cultures were incubated at 37 °C in a humidified 5% CO_2_ incubator for 7 days. Virus-containing cell culture supernatants were aliquoted and stored at −80 °C. Virus titrations of the viral stock and clinical samples were performed on primary swine alveolar macrophages as previously described with minor modifications [18]. PAM cells were seeded in 96-well plates one day before the experiment. A ten-fold dilution of viral stock or clinical samples were prepared in triplicates and transferred onto PAM cells. The presence of virus was visualized by hemadsorption, confirmed by immunofluorescence assay, and titers were calculated using the Spearman–Karber method. The limit of detection for virus isolation in EDTA blood by hemadsorption was 3.41 × 10^3^ HAD_50_/mL and by immunofluorescence 3.41 × 10^2^ TCID_50_/mL. The limit of detection for virus isolation in oropharyngeal (OP) swabs for both hemadsorption and immunofluorescence was 3.41 × 10^1^ TCID_50_ or HAD_50_/mL. The challenge material was prepared by diluting PAM-propagated ASFV-MNG19 viral stock to 360 HAD_50_/mL in Dulbecco’s Modified Eagle Medium (DMEM).

### 2.2. Next Generation Sequencing (NGS)

ASFV-MNG19 DNA extracted from the original virus stock and challenge inoculum were sequenced on the Illumina MiSeq sequencing platform (Illumina; San Diego, CA, USA). The Illumina Nextera XT Library Prep Kit (Illumina; San Diego, CA, USA) was used for the generation of sequencing libraries following the manufacturer’s protocol. Libraries were paired-end sequenced using the Miseq v2 Reagent kit (300 cycles; Illumina; San Diego, CA, USA). Reads were parsed into individual FASTQ files and imported into CLC Genomics Workbench 20 (Qiagen; Carlsbad, CA, USA) for downstream analysis. Reads were trimmed and filtered for removal of the low-quality reads with a Q-score ≤ 25 and reads shorter than 50 nucleotides. The de novo assembly module within CLC Genomic Workbench was used to assemble one near full length contig of 173.5 kB (average coverage of 156.14×) and another of 14.3 kB (average coverage: 142.91×) for the original ASFV-MNG19 stock. The contigs were mapped to the Georgia/2007 reference genome a consensus sequence was generated. The ASFV-MNG19 reference genome was uploaded to GenBank under accession number: OP467597. The sequencing reads for the ASFV-MNG19 challenge material were mapped to the ASFV-MNG19 reference genome using CLC Genomics Workbench to assess any changes within the consensus sequence following passage on PAM cells. The sequence was uploaded to GenBank accession number: OP909713. BLAST [19,20] search using the NCBI nonredundant nucleotide database was completed to identify highest sequence similarity. The complete genomes of 31 ASFV strains including the ASFV-MNG19 original stock and challenge material were aligned with CLC Genomics Workbench. Phylogenetic trees were constructed from the alignment using the maximum likelihood method with 1000 bootstrap replicates using MEGA11 [21]. Additionally, we compared our ASFV-MNG19 genomic consensus sequence to partial ASFV genome sequences related to genes p72 (MT851928 to MT851931), p54 (MT851924 to MT851927), B602L (MT851932 to MT851935), CD2v (MT851936 to MT851938), and intergenic region between I73R and I329L (MT852020 to MT852023), used by Ankhanbaatar et al. [17] for genotyping of the initial Mongolian isolates during the 2019 ASF outbreak. For this particular analysis we only considered the region between the primer loci used by Ankhanbaatar et al. [17] to prevent misidentification of mutations that reside within the primer loci.

### 2.3. Ethics Statement for Use of Animals

All animal studies and experiments were approved and performed under the KSU Institutional Biosafety Committee (IBC, Protocol #: 1314) and the Institutional Animal Care and Use Committee (IACUC, Protocol #: 4265) in compliance with the Animal Welfare Act. The research involving ASFV was conducted in BSL-3 and ABSL-3Ag facilities in the BRI at KSU in Manhattan, KS, USA.

### 2.4. Study Design

**Figure 1:**
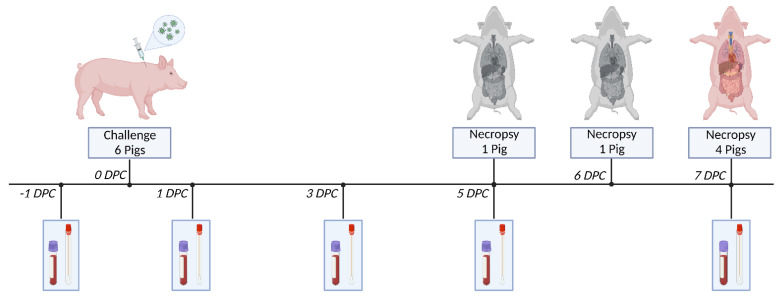
ASFV-MNG19 Challenge Study Schematic. Six crossbred male pigs approximately 2-months-old were challenged with 360 HAD_50_ of ASFV-MNG19. Samples were collected on days −1, 1, 3, 5, and 7 DPC. Samples collected included whole blood (EDTA), OP swabs, and oral fluids collected from chewed ropes at the pen level. Body temperature and clinical scores were assessed daily. One pig (#307) was necropsied on 5 DPC as a result of complications following sample collection. Another pig (#306) was found dead on 6 DPC and subsequently necropsied. The remaining 4 pigs were humanely euthanized and necropsied at 7 DPC due to severe clinical signs.

Clinical disease and progression of ASFV-MNG19 was assessed in six approximately 2-month-old crossbred male pigs (Figure 1). The animals were inoculated intramuscularly (IM) with 1 mL of ASFV-MNG19 (dose of 360 HAD_50_/mL) in the right lateral cervical region cranial to the point of the shoulder. This dose and the IM inoculation route were chosen for consistency with our previous studies to allow for comparison of these data with pigs infected with different ASFV strains. This study does not include other ASFV strains to avoid repetitiveness with our previous studies and reduce the unnecessary use of animals and resources. The IM route provides administration of a controlled dose and produces consistent disease progression. Following inoculation, daily body temperatures and clinical signs were recorded to assess disease progression. Daily health checks were performed every morning by team personnel, as well as in the evening when animals became febrile. Two animals were found dead and the remaining four were humanely euthanized using pentobarbital on 7 days post challenge (DPC) due to severe clinical signs. Necropsies were conducted on all animals, as described below.

### 2.5. Clinical Signs and Samples

Clinical scoring was completed daily and used to assess disease progression and to determine clinical endpoint for severely affected animals to eliminate suffering. The clinical score consisted of 8 criteria which included: liveliness (reluctance to stand or move), body shape (sunken flanks), respiration (tachypnea with abdominal component), neurological signs (ataxia to paralysis), skin (cyanosis and severity), digestive symptoms (diarrhea and vomiting with duration, presence of melena), ocular and nasal discharge, and pyrexia. Each of these criteria were scored from 0 to 3 dependent on severity, with the exception of pyrexia which was scored from 0 to 4; see Supplementary Table S1 for more details. An animal was considered at clinical endpoint when presenting either a cumulative score greater than 10 or severe clinical signs such as neurological signs (paralysis or convulsions), extensive dermal cyanosis, bloody diarrhea, or hypothermia which correspond to the highest score in the respective categories. On clinical sampling days behavior assessments were conducted, which included individual rope chewing, response to OP swabs, and general demeanor. Each pig received an individual score of 0–4 for each category with 0 being normal and 4 uninterested, as detailed in Supplementary Table S2.

Clinical samples were collected on −1, 1, 3, 5, and 7 DPC, consisting of EDTA blood, OP swabs, and oral fluids. The clinical samples were used to follow viremia and viral shedding in bodily fluids over the course of disease using both quantitative real-time PCR and viral titrations.

### 2.6. Quantitative Real-Time PCR Assay for ASFV DNA

ASFV specific DNA was detected and quantified in EDTA-collected blood and OPswabs of challenged pigs by quantitative real-time PCR (qPCR) assay as previously described [22]. Viral DNA from clinical samples was extracted and purified with the MagMax Pathogen RNA/DNA kit (ThermoFisher Scientific; Waltham, MA, USA) using the Kingfisher^TM^ Duo Prime purification system, an automated magnetic bead extraction system, following manufacturer’s protocol with minor modifications. EDTA blood or OP swab supernatant was mixed 1:1 with AL lysis buffer (Qiagen; Carlsbad, CA, USA) and heated at 70 °C for 10 min. Subsequently, 200 µL were mixed with 20 µL of bead mix (equal volume of nucleic acid binding beads and lysis enhancer). The mixture was gently mixed by pipetting and incubated for 2 min at room temperature. Phosphate-buffered saline (PBS, 100 µL) was added to the tube and the entire volume was added to the extraction plate well containing 400 µL lysis buffer, binding buffer and isopropyl alcohol. DNA bound to beads was washed twice with 300 µL of wash solution 1, a third wash was completed with 450 µL wash solution 2, and a final wash with 450 µL of 200 proof molecular grade ethanol. The DNA-bound beads were dried for 5 min prior to being eluted in 100 µL elution buffer. Negative (molecular grade water/PBS) and positive (ASFV p72 plasmid) controls were included with each extraction.

The ASFV DNA extracted from clinical samples was detected and quantified using the primers and probe for the detection of the p72 ASFV gene as previously described by Zsak et al. [23], using a validated protocol performed with the PerfeCTa^®^ FastMix^®^ II (Quanta Biosciences; Gaithersburg, MD, USA) on the CFX96 Touch^TM^ Real-Time PCR Detection System (Bio-Rad; Hercules, CA, USA) as previously described [22]. Duplicate qPCR reactions were performed. Each PCR run included negative and positive controls, which consisted of nuclease-free molecular grade water and ASFV positive amplification control (p72 plasmid), respectively. Due to the analytical limits of the PCR assay, the Ct cutoff was set to 38 for both replicate wells as positive; samples with a single replicate well of 38 or less were classified as suspect. A sample was considered negative if both replicate wells were between 38–45 Ct. The copy number (CN) of ASFV DNA was calculated using a standard curve generated using ten-fold serial dilutions of quantified ASFV p72 plasmid DNA.

### 2.7. Gross and Histological Pathology

Postmortem gross lesions were assessed and scored for all euthanized and found dead pigs. Lesions were scored following the standardization guidelines established by Galindo-Cardiel et al. [24] and previously described by Sunwoo et.al. [22] with modifications. Briefly, gross evaluation was performed on the exterior carcass (dermis, body condition, and eyes), the thoracic cavity (heart and lungs), the abdominal cavity (stomach, liver, gall bladder, kidneys, small intestines, large intestines, and urinary bladder), as well as primary and secondary lymphoid organs including spleen, bone marrow, tonsils, and multiple lymph nodes (mandibular, cervical, tracheobronchial, gastrohepatic, mesenteric, and renal). Additional gross evaluation included oral cavity, trachea, thyroid glands, and the brain. The characterization and classification of gross lesions was performed for each organ. Gross lesions were classified as absent (0), mild (1), moderate (2), and severe (3) with an established numeric scoring system for each organ, see Supplementary Table S3.

Standardized histological evaluation was performed blindly on tonsil, and lymph nodes including submandibular, superficial cervical, perihepatic, mesenteric, and renal, as described by Galindo-Cardiel et al. [24] with minor modifications [22]. Additional tissues evaluated histologically included the lung, liver, spleen, kidney, heart, adrenal glands, skin and gastrointestinal tract. Lesion classification was established as absent (0) or present at mild (1), moderate (2), severe (3) or marked (4) with the numerical score established for tonsils and lymph nodes. Lesion categories scored include: necrosis; cellular or tissue level, fibrin thrombi, deposition in tissue or fibrinoid degeneration of vessels, congestion and hemorrhage, inflammation; infiltrates of macrophages, eosinophils or neutrophils. Specific scoring results for each pig and organ are provided in Supplementary Table S4.

## 3. Results

### 3.1. Whole-Genome Sequencing of the ASFV-MNG19 Isolate

Whole-genome sequencing of the original ASFV-MNG19 isolated from the kidney of an infected pig in the Tov province of Mongolia was performed to confirm viral species and genotype. A near-full-length genome was obtained by de novo assembly. The BLAST search against the NCBI nonredundant nucleotide database show that ASFV-MNG19 is highly similar to other circulating genotype II strains in the region, which includes Chinese, Russian, and Korean strains (Table 1).

The challenge inoculum, ASFV-MNG19 propagated on PAM cells was sequenced and compared with the original ASFV-MNG19 stock prior to use in the described challenge experiment. We detected eight putative mutations in the inoculum compared with the stock (Supplementary Table S5), which could not be reliably confirmed due to the low coverage. Our direct sequencing approach lacks the minimum required depth needed for variant analysis. Targeted sequencing will be necessary to address this. The direct sequencing approach was sufficient for the identification and characterization of the original ASFV-MNG19 stock and challenge inoculum.

**Figure 2:**
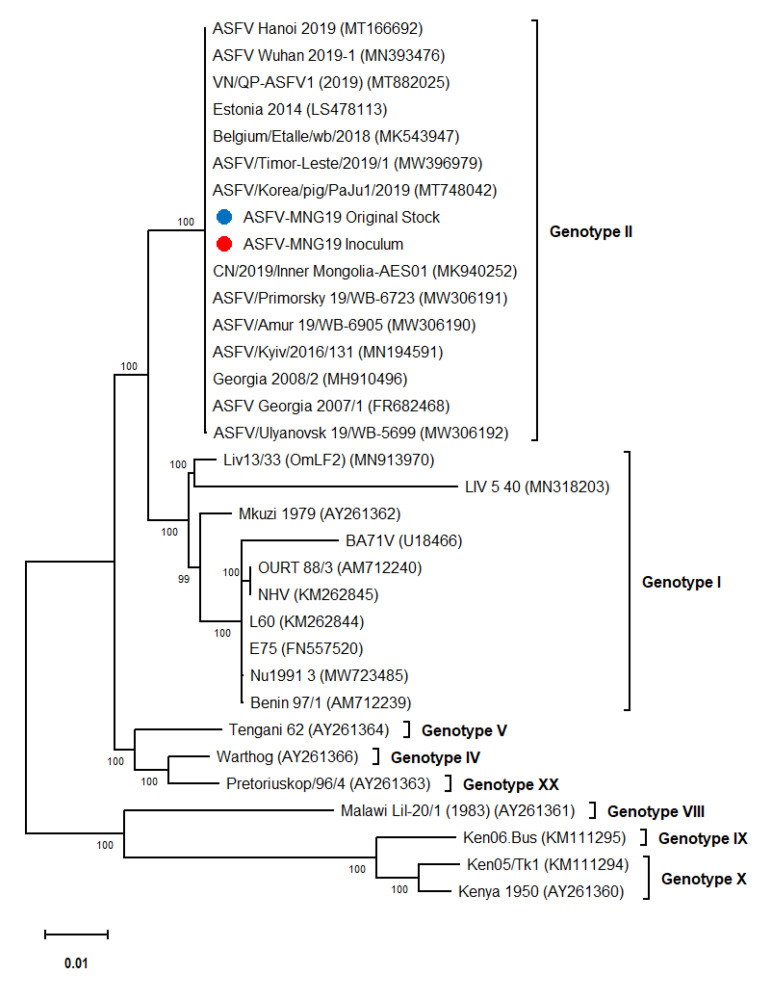
Phylogenetic tree of whole-genome sequences of ASFVs constructed using maximum likelihood approach with 1000 bootstrap replicates. The blue and red dots indicate the ASFV-MNG19 original stock and inoculum used in this challenge study, respectively.

Phylogenetic analysis of the full-length sequences of ASFV-MNG19 isolates used in this study indicated that these viruses clustered with other genotype II isolates detected in Eastern Europe, Russia, and Southeastern Asia (Figure 2), which is in accordance with the global spread of the disease. The comparison of our ASFV-MNG19 isolates revealed a 100% nucleotide identity for the partial p72, full p54, partial B602L, partial CD2v and the intergenic region of I73R and I329L genes of the previously sequenced Mongolian isolates [17]. The ASFV-MNG19 isolates contain the same CVR profile and display the major genotype II genetic features described by Ankhanbaatar et al. [17]. We conclude that our ASFV-MNG19 isolate is a genotype II virus and displays the major genetic features described for the previously genotyped Mongolian isolates.

### 3.2. Clinical Observations following Challenge with ASFV-MNG19 Isolate

**Figure 3:**
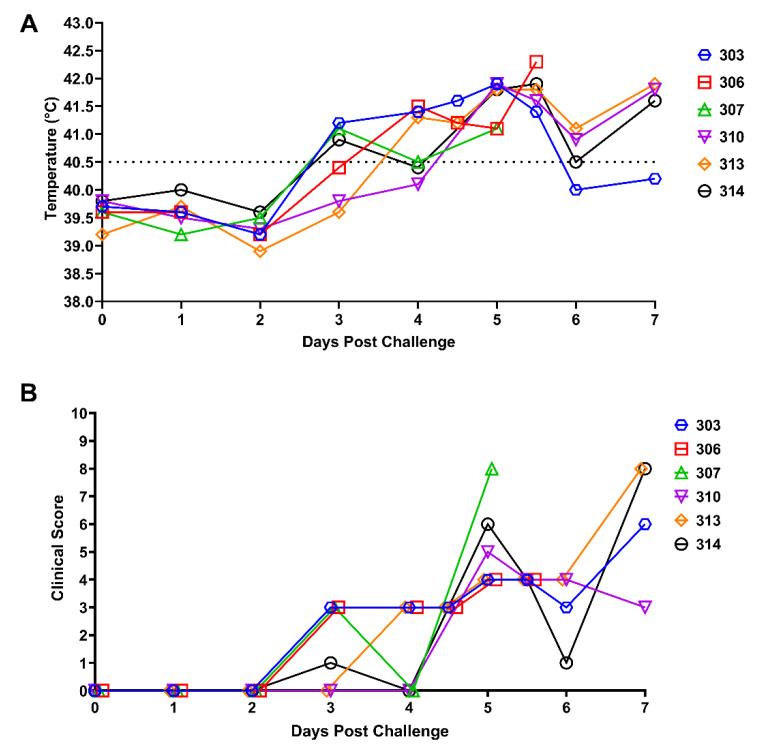
Clinical Observations of ASFV-MNG19 Infected Pigs. Daily body temperatures (**A**) and cumulative clinical score (**B**) of the 6 pigs challenged IM with ASFV-MNG19 from 0 DPC to 7 DPC. Data indicates individual values ((**A**) expressed as °C, (**B**) expressed as an integer value). Data for individual animals are shown. The dashed line in 3A indicates 40.5 °C which is considered a fever for this study.

Following intramuscular inoculation of six pigs with 360 HAD_50_ of the ASFV-MNG19 isolate, the body temperatures were monitored daily or twice daily once temperatures became >40.5 °C (Figure 3A). All six pigs maintained normal temperatures for one or two days post challenge (DPC); by 3 DPC, three of the six pigs had temperatures greater than 40.5 °C. All pigs were febrile by 5 DPC with temperatures greater than 41 °C. The majority of the pigs maintained elevated temperatures until the animals were euthanized due to severe clinical signs or succumbed to ASF. Two animals (#306 and #307) died from an acute form of ASF. Following sample collection at 5 DPC, animal #307 went into respiratory distress followed by cardiovascular collapse; resuscitation was unsuccessful. Animal #306 was found dead at 6 DPC. The remaining four animals were euthanized on 7 DPC due to severe clinical signs (Figure 3B).

Clinical scores were assessed daily along with body temperatures to assess severity of clinical symptoms. The scoring system consisted of eight criteria which included liveliness, body condition, respiration, neurological signs, skin, digestive symptoms, ocular/nasal discharge, and pyrexia. Total clinical scores greater than or equal to 10 were considered clinical endpoints. Daily clinical scores are shown in cumulative form in Figure 3B. Clinical signs following challenge with ASFV-MNG19 were observed starting by 3 DPC when animals became febrile and progressively worsened both in severity and ASF-specific clinical signs. As the pyrexia persisted, animals became lethargic and anorexic, exhibiting reduced interest in rope chewing and normal activities. Two of the animals had respiratory signs consisting of open mouth breathing with an abdominal component following sample collection on 3 DPC; these included #306 which recovered, and #307 which died at 5 DPC as described above. Later in the study, two animals had sunken flanks suggestive of an empty stomach. The remaining four pigs on 7 DPC were displaying mild neurological signs which included stumbling and swaying gaits, and were euthanized on that day. By the end of the study on 7 DPC the major clinical symptoms observed were severe pyrexia, lethargy, and mild neurological and digestive signs. The respiratory signs described were noted following sample collection which could have been exacerbated by excitation from manipulation.

### 3.3. Detection and Quantitation of ASFV DNA by qPCR

**Figure 4:**
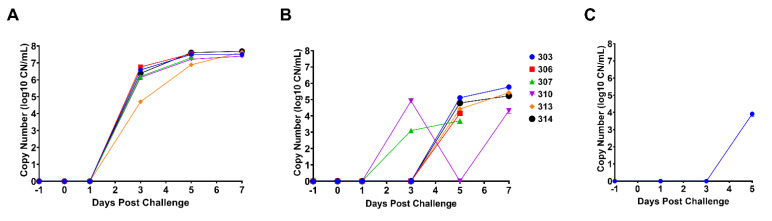
Quantitative real-time PCR for ASFV DNA detected in blood, OP swabs, and oral fluids. (**A**) The calculated copy number (CN) of ASFV DNA extracted from EDTA blood collected on −1, 1, 3, 5, and 7 DPC using a ten-point standard curve. Datapoints for individual pigs are represented. The single data point at 6 DPC represents blood collected from pig #306, which was found dead. (**B**) The calculated copy number of ASFV DNA extracted from OP swabs collected on −1, 1, 3, 5, and 7 DPC using a ten-point standard curve. Datapoints for individual pigs are represented. (**C**) The calculated copy number of ASFV DNA extracted from oral fluids (rope chewing) collected on −1, 1, 3, and 5 DPC using a ten-point standard curve. Datapoints represent the pen level.

Quantitative real-time PCR for detection of ASFV p72 (B646L gene) DNA was performed on viral DNA extracted from EDTA whole blood collected at −1, 1, 3, 5, 6, and 7 DPC; the one blood sample from 6 DPC was collected from pig #306 which was found dead. At 3 DPC, pigs infected with the ASFV-MNG19 isolate had on average 2.49 × 10^6^ CN/mL (Ct = 24.85) of viral DNA present in their blood. Viral loads increased by a log_10_ at 5 DPC and plateaued by 7 DPC (Figure 4A). The increasing viral loads correlated with the prolonged, severe pyrexia and increased clinical scores. Viral DNA was detected in OP swabs at 5 DPC from all animals, except pig #310, which had detectable viral DNA at 3 DPC but not at 5 DPC as a result of transient shedding or sampling technique (Figure 4B). The remaining animals that were euthanized on 7 DPC had detectable ASFV DNA in their OP swabs. This is indicative of virus shedding in saliva and is corroborated further by detectable viral DNA in oral fluids collected at the pen level on 5 DPC (Figure 4C), where 3/6 pigs were observed chewing the ropes but with reduced enthusiasm, see Supplementary Table S6.

### 3.4. Viral Titrations in Blood and OP Swabs

**Figure 5:**
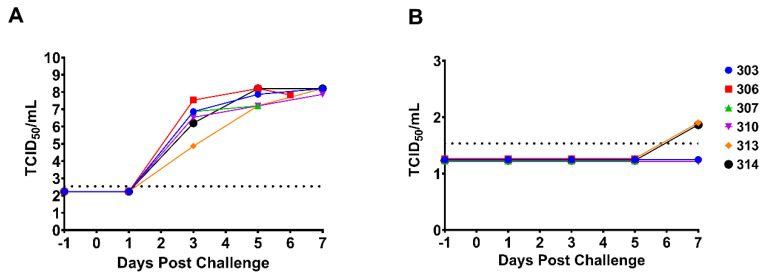
Viral titers in blood and OP swabs. (**A**) The log transformed viral titers (TCID_50_/mL) of ASFV-MNG19 in EDTA blood collected on −1, 1, 3, 5, and 7 DPC. All samples were run in triplicate. The datapoints represent the calculated titers using the Spearman–Karber method. The dashed line represents the limit of detection for the assay. Datapoints for individual pigs are represented. The single data point at 6 DPC represents samples collected from pig #306 that was found dead. (**B**) The log transformed viral titers (TCID_50_/mL) of ASFV-MNG19 in OP swabs collected on −1, 1, 3, 5, and 7 DPC. All samples were run in triplicate. The datapoints represent the calculated titers using the Spearman–Karber method. The dashed line represents the limit of detection for the assay. Datapoints for individual pigs are represented.

Virus titrations were performed on the EDTA blood and OP swabs collected at −1 to 7 DPC for all animals. The presence of infectious virus was determined by hemadsorption and confirmed by immunofluorescence assay. As expected, there was no detectable virus at −1 DPC in blood or swabs. All animals had detectable infectious virus in the blood by 3 DPC with titers ranging from 10^4^–10^6^ TCID_50_/mL. As disease progressed, virus titers in the blood increased to 10^7^–10^8^ TCID_50_/mL (Figure 5A). The viral titers obtained from blood samples were consistent with the viral DNA loads determined by qPCR (see Figure 4A). No infectious virus was detected in the OP swabs until 7 DPC; only low levels (7.34 × 10^1^ TCID_50_/mL) were detected from 2/6 pigs (Figure 5B). However, qPCR detected viral DNA at 5 DPC from 5/6 pigs.

### 3.5. Gross Pathologic Lesions

**Figure 6:**
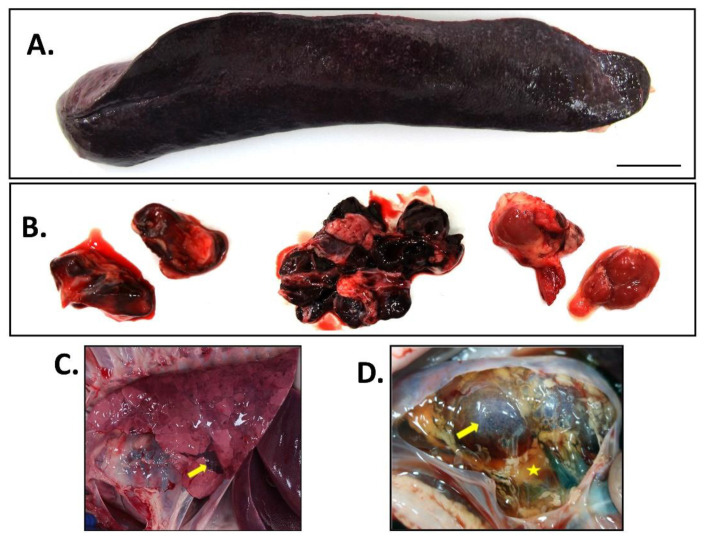
Gross pathology lesions following ASFV-MNG19 infection. (**A**) Spleen: severe splenomegaly; (**B**) Lymph nodes: hemorrhage (from left to right: renal lymph node, gastrohepatic lymph node, and cranial mediastinal lymph node); (**C**) Lungs: pulmonary edema with congestion, consolidation (yellow arrow), and ecchymosis; (**D**) Kidney: retroperitoneal serous effusion indicated by the yellow star, petechial hemorrhages of renal cortex indicated by the yellow arrow.

All pigs found dead or euthanized due to ASF were necropsied to evaluate gross pathologic lesions as described in Supplementary Table S3. All pigs displayed loss in body condition with mild to moderate cutaneous hyperemia of the skin at the ear tips, ventral abdomen and distal limbs. Moderate to severe gross lesions consistent with acute ASF included splenomegaly, hemorrhagic lymph nodes, and pulmonary congestion and edema. Splenic congestion and necrosis were consistently observed (Figure 6A). Lymph nodes were moderately enlarged with ranging severity of edema (mandibular and prescapular lymph nodes) or hemorrhages. Severe hemorrhagic lymphadenopathy was observed in the cranial mediastinal, renal, and gastrohepatic lymph nodes in 4/6 pigs (Figure 6B). All pigs had moderate to moderate-to-severe pulmonary congestion and edema, and two of the pigs had multifocal pulmonary hemorrhage with consolidation (Figure 6C). Mild-to-moderate renal corticomedullary congestion was noted in 4/6 pigs, renal cortical thrombosis was observed in 3/6 pigs. One pig had marked retroperitoneal effusion with a viscous serous fluid filling, expanding to the connective tissue of the caudal dorsal abdomen and surrounding the kidneys (Figure 6D). Gross lesions observed for the cardiovascular system consisted of perivascular hemorrhages at venipuncture site for 2/6 pigs reflective of ASF induced coagulopathy. Thrombi were observed in the interventricular branch of the right coronary artery and the right ventricle of the heart of one pig, further supporting the ASF-associated coagulopathy. Moderate locally extensive hyperemia of the epicardium was observed in the auricles of the right and left atria in 3/6 pigs. Other ASF associated lesions observed were severe diffuse subarachnoid hemorrhagic meningitis (5/6 pigs), moderate hyperemic gastritis (5/6), and mild to moderate hemorrhagic colitis (5/6). The average gross score for all six pigs was 44.50 ± 13.19 which is considered moderate acute ASF; detailed lesion scoring for each system are described in Supplementary Tables S7 and S8.

### 3.6. Histopathological Lesions

**Figure 7:**
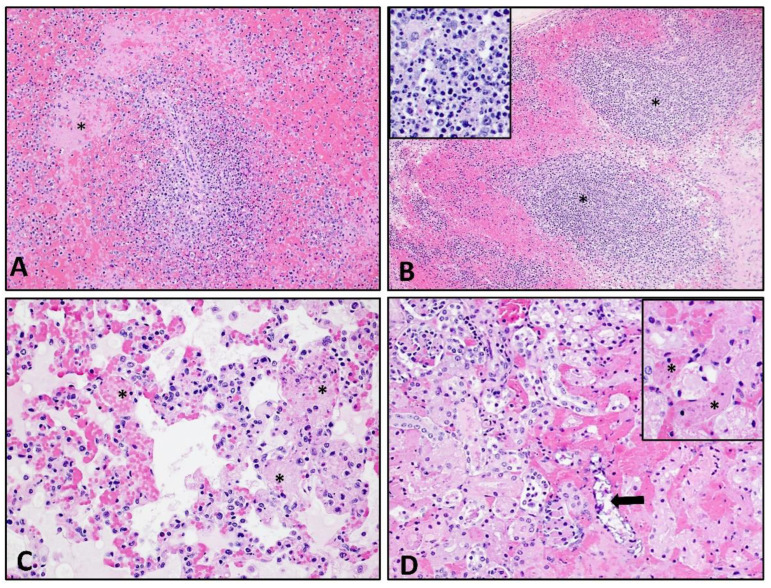
Histological lesions following ASFV-MNG19 infection. (**A**) Spleen: Marked lympholysis and collapse of periarterial sheaths and fibrin thrombi (asterisk) in marginal zone (100×). (**B**) Renal lymph node: Marked lympholysis and necrosis (asterisk and insert) with loss of cortical and sinusoidal lymphocytes accompanied by marked filling and expansion of the subcapsular and medullary sinuses with hemorrhage and fibrin (100× and 400× insert). (**C**) Lung: Marked expansion of the alveolar spaces by congestion, fibrin thrombi (asterisk) and necrotic cellular debris, eosinophilic proteinaceous material; edema, fibrin and occasional macrophages fill the alveolar spaces (200×). (**D**) Kidney: Multifocal congestion and fibrin thrombosis (insert-asterisk) of the interstitial capillaries accompanied by hemorrhage and coagulative necrosis and dystrophic mineralization (arrow) of tubules (200×, 400×-insert).

Histological analysis was performed on liver, lung, kidney, spleen, and tonsil, as well as mesenteric, mandibular, renal, gastrohepatic, prescapular, ileocecal and inguinal lymph nodes. Figure 7 contains representative images of histopathological changes seen in the spleen (Figure 7A), lymph node (Figure 7B), lung (Figure 7C), and kidney (Figure 7D) from pigs infected with ASFV-MNG19. Changes in the spleen and lymph nodes consisted of marked lympholysis with collapse and loss of periarterial sheaths/follicles, accompanied by fibrin clots in the marginal zone of the spleen and sinuses of the lymph nodes. Fibrinoid degeneration and vasculitis is prominent in many lymph nodes and is frequently accompanied by subcapsular and sinusoidal hemorrhage (Figure 7B). Within the lungs there was moderate to severe congestion of alveolar capillaries which commonly contained fibrin thrombi, cellular debris and large foamy macrophages. Edema, fibrin and macrophages commonly filled alveolar spaces and were occasionally accompanied by hemorrhage (Figure 7C). Figure 7D depicts renal lesions seen in two pigs with marked systemic intravascular coagulation. There were large fibrin thrombi filling interstitial capillaries, which were multifocal throughout the cortex resulting in coagulation necrosis and dystrophic mineralization of tubules. Fibrin thrombi occurred in glomeruli. Multifocal areas of hemorrhage occurred in the cortex and interstitium, frequently occurring around large vessels. Supplementary Table S4 contains the scoring of individual lymph nodes for all infected pigs and ranking of the lesions for each lymph node. Overall, two pigs in the group had severe lymphoid lesions, while three were classified with moderate lymphoid lesions and one with mild lymphoid lesions. The gastrohepatic and the renal lymph nodes were most severely affected followed by the ileocecal, mandibular and cranial mediastinal lymph nodes, see Supplementary Table S4.

## 4. Discussion

The results in this study show that the ASFV strain isolated from naturally infected pigs during the 2019 outbreak in Mongolia is a genotype II ASFV strain with high similarity to regional genotype II ASFV strains identified in 2018 and 2019 from China, Korea, and Russia. Following passage of ASFV-MNG19 on PAM cells several putative mutations were identified in the consensus sequence, which could not be reliably validated due to low coverage in these regions. A targeted whole-genome sequencing approach is required to determine if these mutations are adaptations arising from passage on primary cell lines or sequencing artifacts. The sequence of the ASFV-MNG19 strain was compared with the available partial sequences from an isolate collected in the Bulgan province of Mongolia during the 2019 outbreak and found to be identical to these partial sequences. Since only partial sequences are available for the Bulgan isolate, we cannot confidently determine that these two isolates are the same strain. Further studies are needed to infer the relationship between these two isolates and others, which include whole-genome sequencing of the Bulgan isolate and other isolates collected during the 2019 outbreak.

The intramuscular inoculation of pigs with a 360 HAD_50_ dose of ASFV-MNG19 resulted in rapid, fatal disease, with all animals dying or having to be euthanized due to severe clinical disease by 7 DPC. Clinical signs consisting of lethargy and pyrexia were observed by 3 DPC, which continued to progress, and by 5 DPC all animals developed high grade fevers (≥41 °C) with progressively worsen clinical signs. Viremia with virus titers of 10^6^ TCID_50_/mL by 3 DPC, which rose to 10^8^ TCID_50_/mL by 5 or 7 DPC, correlated with presentation of clinical signs. Two of the animals died suddenly on 5 and 6 DPC, respectively, within 2 days of developing clinical signs. The ASFV-MNG19 strain efficiently replicates and disseminates systemically in domestic pigs, resulting in severe disease within days after clinical signs are noted. Gross lesions observed at necropsy were consistent with acute ASF such as splenomegaly, enlarged hemorrhagic lymph nodes, and pulmonary edema. Similar to what was seen with other Eurasian ASFV genotype II strains in domestic pigs, the most severely affected lymph nodes with gross and histological lesions were the gastrohepatic and the renal lymph nodes followed by the ileocecal, mandibular and cranial mediastinal lymph nodes [25,26]. The clinical signs, gross lesions, and sudden death observed shortly after development of clinical symptoms were in line with what has been described for field infections with Mongolia isolates [17]. Based on these findings we have found no evidence that the virulence of ASFV-MNG19 was altered following propagation on primary porcine cells.

As demonstrated previously with other ASFV strains, the ASFV-MNG19 strain most likely can be transmitted through oral secretions [25,27,28,29]. In the current study, infectious virus was detected in the OP swabs of two pigs at 7 DPC. We were unable to determine if the animals continued to shed virus since all were euthanized at 7 DPC. The detection of infectious virus in OP swabs suggests the virus could be shed and potentially transmitted through oral secretions. However, one of the limitations of this study is the lack of naïve contact control pigs to study transmission.

The data from this study provides information on the genetics, virulence and virus–host infection dynamics of the genotype II ASFV-MNG19 field isolate. The present study confirms that the ASFV-MNG19 strain is a virulent genotype II virus that causes similar clinical disease presentation and progression as other historic and regionally circulating genotype II ASFV isolates. Infection of pigs with the ASFV-MNG19 isolate resulted in rapid progression of clinical disease resulting in death from acute ASF. The incubation period of 3–5 days and clinical disease progression are similar to other genotype II strains, which include regional strains such as a 2018 Chinese isolate [29] and a 2019 Vietnamese isolate [27] as well as the historic genotype II Georgia2007/1 [25] and Armenia 2008 [30] isolates, which all cause acute ASF after IM inoculation.

The global ASF status has drastically changed over the past 15 years beginning with the 2007 outbreak in the country of Georgia allowing ASFV to spread across eastern Europe and Russia with outbreaks reported between 2008 and 2017 in domestic pig and wild boar populations [12,13,14,31,32]. By 2018, ASFV had reached China causing a devastating outbreak that resulted in the loss of 1.2 million pigs and massive economic losses [33]. Afterwards, ASFV continued to spread to neighboring countries and regions [15] as well as globally with outbreaks in India [34] and Germany in 2020 [35], and in the Dominican Republic in 2021 [28,36]. Some of these outbreaks were resolved through strict mitigation strategies that included quarantine, restrictions on animal movement, and depopulation of affected herds and/or wild boar. All of these outbreaks are the result of infections with virulent genotype II ASFV strains [15,17,27,28]. It is important that controlled experimental studies are conducted to evaluate the genetics, virulence and clinical progression of emerging ASFV strains described in the field, and to aid in the development and validation of diagnostic tools used by regulatory agencies for the rapid detection and implementation of mitigation strategies.

## Figures and Tables

**Table 1:** The ten sequences with the highest similarity to ASFV-MNG19.

Accession	Name	Percent Identity
MK128995.1	China/2018/AnhuiXCGQ	99.99
MN172368.1	ASFV/pig/China/CAS19-01/2019	99.99
MK333180.1	Pig/HLJ/2018	99.99
ON075797.1	Korea/YC1/2019	99.98
MW306191.1	ASFV/Primorsky 19/WB-6723	99.99
MW396979.1	ASFV/Timor-Leste/2019/1	99.98
MN715134.1	ASFV_HU_2018	99.98
OL692744.1	IND/AR/SD-61/2020	99.98
OL692743.1	IND/AS/SD-02/2020	99.98
MW049116.1	ASFV Korea/pig/Yeoncheon1/2019	99.98

BLAST search results for the ten ASFV genomes with the highest sequence similarity to the ASFV-MNG19 full genome. First is NCBI accession number, followed by sequence name, and percent identity.

## Data Availability

Not applicable.

## Supplementary Materials

The following supporting information can be downloaded at: https://www.mdpi.com/article/10.3390/v14122698/s1, Table S1: Clinical signs and scoring parameters; Table S2: Behavior assessment and scoring parameters; Table S3: Evaluation criteria for gross pathology and scoring; Table S4: Histological lesion score for lymphoid tissues of ASFV-MNG19 challenged animals; Table S5: Identified mutations between ASFV-MNG19 original stock and challenge inoculum; Table S6: Behavior assessment scores; Table S7: Gross lesion scores for ASFV-MNG19 infected animals; Table S8: Gross lesion score for lymphoid tissues of ASFV-MNG19 infected animals.
