# Supplementary material for: Experimental Infection of Domestic Pigs with African Swine Fever Virus Isolated in 2019 in Mongolia
Source: Viruses. 2022 Dec 1;14(12):2698. doi: 10.3390/v14122698 (PMC9781604; doi:10.3390/v14122698)
Supplement: Supplementary file 1 [file viruses-14-02698-s001.zip › viruses-1985950-supplementary.pdf]

Table S1. Clinical signs and scoring parameters. Previously described by Sunwoo et al. [22].

Clinical Signs	Severity (scoring)				
	**0**	**1**	**2**	**3**	**4***
Liveliness	No Abnormality	Reduced liveliness, stands without help	Sluggish, stands with help	Dormant, refusal to stand with or without help	Not Used
Body Shape	No Abnormality	Empty Stomach (sunken flanks)	Empty Stomach with weight loss indicators	Wasting (ribs, vertebrae visible, rough hair)	Not Used
Breathing	No Abnormality	Increased Respiratory Rate	Significantly increased respiratory rate, abdominal breathing	Difficult Breathing (open mouth breathing, wheezing, coughing)	Not Used
Neurological Signs	No Abnormality	Stumbling gait, quickly corrected	Ataxia/paresis of the hindquarters, continues to walk	Paralysis of the hindquarters, unable to stand, convulsions	Not Used
Skin	No Abnormality	Red skin, cyanosis of the skin (<10%)	Cyanosis of the skin (10-25%), occasional skin bleeding	Cyanosis of the skin (>25%), large blue/black spots, cold skin, large subcutaneous bleeding, skin necrosis/ulceration	Not Used
Digestive Symptoms	No Abnormality	Diarrhea (<24 Hours)	Diarrhea (>24 hours), occasional vomiting	Bloody diarrhea and frequent vomiting	Not Used
Ocular/Nasal Discharge	No Abnormality	Thin discharge from nose and/or eyes (w/o admixtures)	Thick discharge from nose and/or eyes (no blood)	Bloody discharge from nose and/or eyes	Not Used
Fever	37.8-40.5˚C	≥40.5˚C, <41 ˚C	≥40.5˚C for two sequential days, <41 ˚C	≥41˚C	< 37.8˚C

*: This clinical score is only used in the fever category because it constitutes immediate euthanasia.

Table S2. Behavior assessment and scoring parameters.

Behavior Assessment	Scoring				
	**0**	**1**	**2**	**3**	**4**
Rope Chewing	Participating exuberantly	Participation is active but drops off more quickly	Participation starts slow and is poor	Refused to chew for longer than 5-10 seconds but shows interest in other activities	Not engaged, not participating
Individual Swab	Allows collection but protests as a normal pig	Allows collection with minimal protest	Allows collection but has moderately reduced liveliness	Not engaged seems unaware of the activity	Not used
General Demeanor	Normal: inquisitive, responsiveness to voice, wants to play	Initially is interested but drops off quickly	Interest starts slow and is poor	Interest is very poor; markedly reduced and short lived	Not engaged, seems uninterested in activities; reluctant to move

Table S3. Evaluation criteria for gross pathology and scoring. Scoring was modified from Galindo-Cardiel et al [24] and Sunwoo et al. [22].

System	Evaluation criteria and score
Body Condition	1 category scored: Normal (0); Mild (1), vertebrae and pelvic bones detectable by firm palpation and ribs not overtly visible but palpable; Moderate (2), vertebrae and pelvic bones visible and prominent ribs visible; Severe (3), vertebrae and pelvic bone obvious with muscle wasting, prominent ribs.
Integument	1 category: Normal (0); pronounced hyperemia; Mild (1); petechia/ecchymosis; Moderate (2); cyanosis/infarction; Severe (3).
Cardiovascular	2 categories scored*: a) Systemic evidence of hemorrhage, edema, and/or thrombosis of various organ systems such as: subcutaneous/intramuscular/abdominal/central nervous system/body cavity; b) heart: epicarditis, thrombosis or pericardial effusion.
Liver	2 categories scored*: a) Hepatopathy including enhanced lobular pattern, intrahepatic mottling, congestion, friable parenchyma; b) biliary tree: gall bladder, common bile duct and vascular hilus, edema and/or hemorrhage with prominent vasculature.
Lung	3 categories scored*: a) Swelling and edema; b) congestion and/or hemorrhage; c) pneumonia/consolidation characterized by pulmonary collapse and a lack of it leading to rib impressions.
Spleen	2 categories scored*: a) Congestion and/or hemorrhage; b) necrosis or infarction.
Lymph nodes/tonsil	2 categories scored*: a) Edema and/or congestion; b) hemorrhage.
Kidney	1 category scored*: Congestion, edema, hemorrhage, and/or thrombosis (multifocal, cortical, medullary, and/or subcapsular).
Gastrointestinal	5 categories scored**: a) Gastric hyperemia and/or ulceration; b) mucosal edema, hemorrhage, and/or necrosis of the intestinal tract; c) diarrhea; d) hemorrhagic diarrhea; e) ascites, fibrin, and/or peritonitis.

*: Each category was scored as normal (0), mild (1), moderate (2), or severe (3).

**: Category (a) is scored as normal (0), mild (1), moderate (2), or severe (3). Category (b) is scored as mild to moderate (1) and severe or marked (2). Categories (c-e) are scored as one point if present regardless of severity.

Note: Each category is scored as described above (in table S3) and a total sum per animal is compiled to provide analytical interpretation of gross lesions. Total gross scores less than 30 represent overall mild gross ASFV lesions, 31-47 represent overall moderate gross lesions, while scores 48 or greater represent severe gross ASFV lesions. The maximum total score per pig is 84.

Table S4: Histological lesion score for lymphoid tissues of ASFV-MNG19 challenged animals.

Organ	Criteria	Pig ID #						Mean Score	Standard Deviation	95% Confidence Interval
		**303**	**306**	**307**	**310**	**313**	**314**			
Mesenteric Lymph Node		1	6	3	3	2	5	3.33	1.86	2.31
	Necrosis	1	3	1	1	1	2			
	Fibrin/clots	0	1	1	1	0	2			
	Hemorrhage	0	1	0	0	0	0			
	Inflammation	0	1	1	1	1	1			
Mandibular Lymph Node		6	8	3	4	7	9	6.17	2.32	2.88
	Necrosis	3	3	1	1	2	3			
	Fibrin/clots	2	3	1	1	2	3			
	Hemorrhage	1	1	0	1	2	1			
	Inflammation	0	1	1	1	1	2			
Tonsil		10	7	2	2	2	10	5.50	3.99	4.95
	Necrosis	3	3	1	1	1	3			
	Fibrin/clots	3	2	0	0	0	3			
	Hemorrhage	1	1	0	1	0	2			
	Inflammation	3	1	1	0	1	2			
Gastrohepatic Lymph Node		12	10	5	8	7	12	9.00	2.83	3.51
	Necrosis	3	3	1	1	2	3			
	Fibrin/clots	3	2	1	2	1	3			
	Hemorrhage	4	3	2	4	3	4			
	Inflammation	2	2	1	1	1	2			

Renal Lymph Node		8	9	7	6	7	9	7.67	1.21	1.50
	Necrosis	2	3	3	1	2	3			
	Fibrin/clots	2	2	2	1	1	2			
	Hemorrhage	3	3	1	3	3	3			
	Inflammation	1	1	1	1	1	1			
Cranial Mediastinal Lymph Node		6	12	1	8	6	8	6.83	3.60	4.47
	Necrosis	2	4	1	3	2	3			
	Fibrin/clots	2	3	0	3	1	3			
	Hemorrhage	1	4	0	1	2	0			
	Inflammation	1	1	0	1	1	2			
Prescapular Lymph Node		6	8	1	1	7	11	5.67	3.98	4.95
	Necrosis	1	4	1	1	3	4			
	Fibrin/clots	1	2	0	0	1	4			
	Hemorrhage	1	1	0	0	1	0			
	Inflammation	3	1	0	0	2	3			
Inguinal Lymph Node		7	3	1	1	8	11	5.17	4.12	5.11
	Necrosis	1	2	1	1	2	4			
	Fibrin/clots	3	1	0	0	3	4			
	Hemorrhage	2	0	0	0	1	0			
	Inflammation	1	0	0	0	2	3			
Ileocecal Lymph Node		7	7	1	10	11	6	7.00	3.52	4.37
	Necrosis	3	3	1	3	3	2			
	Fibrin/clots	3	2	0	2	3	1			
	Hemorrhage	0	1	0	4	3	0			
	Inflammation	1	1	0	1	2	3			
Total score***		63	70	24	43	57	81			

***Total possible score for each lymph node is 16 and the total possible score is 144. Lesions are ranked as mild: < 30, moderate: 31-60, severe: 61-80, marked: 80+.

Table S5: Identified mutations between ASFV-MNG19 original stock and challenge inoculum.

Position	ASFV-MNG19 Original Stock	ASFV-MNG19 P1 PAM	Amino Acid Change	Gene
36244-36245	GC	AA	Q281K	MGF505-4R
36268	G	A	Synonymous	MGF505-4R
36272	G	A	E290K	MGF505-4R
105225	C	A	N11K	B125R
105229	G	A	E13K	B125R
110321	C	A	Synonymous	G1340L
110329	T	A	N809Y	G1340L

Table S6. Behavior assessment scores.

Pig ID #	Criteria	DPC		
		1	3	5
**303**		**0**	**0**	**4**
	**Rope Chewing**	0	0	2
	**Individual Swab**	0	0	0
	**General Demeanor**	0	0	2
**306**		**0**	**0**	**2**
	**Rope Chewing**	0	0	1
	**Individual Swab**	0	0	0
	**General Demeanor**	0	0	1
**307**		**0**	**0**	**8**
	**Rope Chewing**	0	0	4
	**Individual Swab**	0	0	0
	**General Demeanor**	0	0	4
**310**		**0**	**0**	**8**
	**Rope Chewing**	0	0	4
	**Individual Swab**	0	0	0
	**General Demeanor**	0	0	4
**313**		**0**	**0**	**4**
	**Rope Chewing**	0	0	2
	**Individual Swab**	0	0	0
	**General Demeanor**	0	0	2
**314**		**0**	**0**	**8**
	**Rope Chewing**	0	0	4
	**Individual Swab**	0	0	0
	**General Demeanor**	0	0	4

Table S7: Gross lesion scores for ASFV-MNG19 infected animals.

Organ System	303 Lesions (score)	306 Lesions (score)	307 Lesions (score)	310 Lesions (score)	313 Lesions (score)	314 Lesions (score)
**Body Condition**	Prominent ribs and pelvis, moderate loss of body condition; (2)	Mild loss of body condition with muscle atrophy; (1)	Sunken flanks and ribs are prominent; (2)	Mild loss of body condition; (1)	Mild loss of body condition with muscle atrophy; (3)	Moderate loss of body condition with muscle atrophy; (3)
**Integument**	Ventrally diffuse moderate ecchymosis; (2)	Mild cutaneous hyperemia; (1)	Multifocal cutaneous hyperemia; (1)	Ventrally Diffuse moderate ecchymosis; (2)	Mild Multifocal ecchymosis; (2)	Mild Multifocal ecchymosis; (2)
**Cardiovascular**	Mild focal fibrinous pericarditis of left ventricle; (2)	Mild cervical hemorrhage and polyserositis; (4)	Severe cervical hemorrhage with thrombi. thrombi in the subsinusoidal interventricular branch of right coronary artery. Hemorrhagic left auricle; (2)	Moderate hemorrhage of the right atria or auricle. Multifocal fibrous of right ventricle; (1)	Moderate hemorrhage of the left auricle, pericardial effusion; (2)	Mild congestion of interventricular vasculature; (3)
**Liver**	Mild congestion of the gall bladder and bile ducts; (1)	Congested bile ducts; (2)	Normal with a full gall bladder.; (0)	Congestion of hepatic vascular, enlarged gall bladder; (2)	Cholestasis, congested hepatic vascular; (2)	Cholestasis, congest hepatic vasculature; (2)
**Lung**	Multifocal pulmonary hemorrhages, Severe pulmonary congestion with edema and consolidation; (6)	Mild pulmonary edema; (1)	Multifocal to coalescing thrombi, pulmonary congestion and edema; (4)	Moderate to severe pulmonary congestion with edema and consolidation ; (5)	Severe pulmonary edema and multifocal congestion; (5)	Severe pulmonary congestion with edema and consolidation with pneumonia (multifocal to diffuse); (7)
**Spleen**	Moderate hemorrhagic splenomegaly; (3)	Moderate hemorrhagic splenomegaly; (3)	Severe multifocal necrotic hemorrhagic splenomegaly; (5)	Severe hemorrhagic splenomegaly with necrotic foci; (4)	Multifocal hemorrhagic splenomegaly; (3)	Severe hemorrhagic splenomegaly; (5)

**Kidney**	Moderate diffuse corticomedullary congestion; (1)	Focal corticomedullary thrombi; (1)	Mild diffuse corticomedullary congestion ; (1)	Mild diffuse edema; (1)	Moderate corticomedullary congestion, multifocal cortical thrombosis; (2)	Perirenal serous effusion, cortical petechiation; Mild corticomedullary congestion.; (2)
**Gastrointestinal**	Moderate hyperemia of the gastric mucosa. Moderate hemorrhagic colitis, petechiation of the rectal mucosa, ascites, and diarrhea. (7)	Moderate hyperemia of the gastric mucosa. Mild hemorrhagic enteritis, petechiation of the colon and serosanguineous ascites with fibrin and diarrhea; (7)	Mild hyperemic gastric mucosa and diarrhea; (4)	Mild hyperemia of the gastric mucosa. Mild mucosal edema of the small intestines and diarrhea; (3)	Mild hyperemia of the gastric mucosa. Moderate mucosal edema with petechiation of the colon; (5)	Moderate hyperemia of the gastric mucosa. Moderate mucosal edema and necrotic enteritis. Ascites and diarrhea; (9)

Table S8: Gross lesion score for lymphoid tissues of ASFV-MNG19 infected animals.

Lymphoid Tissue	303 Lesions (score)	306 Lesions (score)	307 Lesions (score)	310 Lesions (score)	313 Lesions (score)	314 Lesions (score)
**Mandibular Lymph Node**	Mild lymphadenopathy; (0)	Severe edematous lymphadenopathy; (3)	Normal; (0)	Mild hemorrhagic lymphadenopathy; (2)	Severe hemorrhagic lymphadenopathy; (5)	Mild lymphadenopathy; (2)
**Cranial Mediastinal Lymph Node**	Mild hemorrhagic lymphadenopathy; (2)	Moderate hemorrhagic lymphadenopathy; (4)	Mild edematous lymphadenopathy; (1)	Moderate hemorrhagic lymphadenopathy; (3)	Severe hemorrhagic lymphadenopathy; (5)	Moderate hemorrhagic lymphadenopathy; (4)
**Mesenteric Lymph Node**	Mild lymphadenopathy; (2)	Moderate edematous lymphadenopathy; (2)	Mild edematous lymphadenopathy; (1)	Moderate hemorrhagic lymphadenopathy; (3)	Severe hemorrhagic lymphadenopathy; (5)	Mild lymphadenopathy; (2)
**Renal Lymph Node**	Severe hemorrhagic lymphadenopathy; (6)	Severe hemorrhagic lymphadenopathy; (6)	Mild edematous lymphadenopathy; (1)	Severe hemorrhagic lymphadenopathy; (6)	Severe hemorrhagic lymphadenopathy; (6)	Severe hemorrhagic lymphadenopathy; (6)
**Gastrohepatic Lymph Node**	Severe hemorrhagic lymphadenopathy; (6)	Severe hemorrhagic lymphadenopathy; (6)	Mild edematous lymphadenopathy; (1)	Severe hemorrhagic lymphadenopathy; (6)	Severe hemorrhagic lymphadenopathy; (6)	Moderate hemorrhagic lymphadenopathy; (5)
**Prescapular Lymph Node**	Mild hemorrhagic lymphadenopathy; (2)	Mild lymphadenopathy; (1)	Normal; (0)	Mild hemorrhagic lymphadenopathy; (2)	Moderate lymphadenopathy; (3)	Moderate lymphadenopathy; (3)
**Tonsil**	Normal; (0)	Mild congestion with edema; (1)	Normal; (0)	Mild congestion with edema; (1)	Severe congestion and edema with moderate hemorrhage; (5)	Severe congestion and edema; (3)
**Severity (total score) ***	Moderate; (42)	Moderate; (43)	Mild; (23)	Moderate; (42)	Severe; (59)	Severe; (58)

*: Total gross score includes scores from table S7 and table S8.
